# DOTA-ZOL: A Promising Tool in Diagnosis and Palliative Therapy of Bone Metastasis—Challenges and Critical Points in Implementation into Clinical Routine

**DOI:** 10.3390/molecules25132988

**Published:** 2020-06-30

**Authors:** Michael Meisenheimer, Stefan Kürpig, Markus Essler, Elisabeth Eppard

**Affiliations:** 1 Department of Nuclear Medicine, University Hospital Bonn, D-53127 Bonn, Germany; michael.meisenheimer@ukbonn.de (M.M.); stefan.kuerpig@ukbonn.de (S.K.); markus.essler@ukbonn.de (M.E.); 2 Positronpharma SA, Rancagua 878, Providencia 7500921, Chile

**Keywords:** gallium-68, bone metastasis, radiolabelling synthesis, quality control, module system

## Abstract

The novel compound 1,4,7,10-tetraazacyclododecane-1,4,7,10-tetraacetic acid (DOTA)-ZOL (DOTA-conjugated zoledronic acid) is a promising candidate for the diagnosis and therapy of bone metastasis. The combination of the published methodology for this bisphosphonate with pharmaceutical and regulatory requirements turned out to be unexpectedly challenging. The scope of this work is the presentation and discussion of problems encountered during this process. Briefly, the radiolabelling process and purification, as well as the quality control published, did not meet the expectations. The constant effort setting up an automated radiolabelling procedure resulted in (a) an enhanced manual method using coated glass reactors, (b) a combination of three different reliable radio thin-layer chromatography (TLC) methods instead of the published and (c) a preliminary radio high-pressure liquid chromatography (HPLC) method for identification of the compound. Additionally, an automated radiolabelling process was developed, but it requires further improvement, e.g., in terms of a reactor vessel or purification of the crude product. The published purification method was found to be unsuitable for clinical routine, and an intense screening did not lead to a satisfactory result; here, more research is necessary. To sum up, implementation of DOTA-ZOL was possible but revealed a lot of critical points, of which not all could be resolved completely yet.

## 1. Introduction

The bone is, more than other tissues, affected by the metastasis of solid tumours [1,2] and is a prevalent complication in cancers of breast (BCa), prostate (PCa) and lung (LCa) [3]. PCa metastasise preferentially into bone, which represents a matrix facilitating tumour growth and promoting a vicious cycle between metastasis and bone pathology [4,5]. Of all new diagnosed patients with PCa, 3% already have, and about 11.5% will develop, bone metastases [6]. As shown by an autopsy study, approximately 90% of men dying from PCa suffer from bone metastases [7]. Apart from this, approximately 90% of patients diagnosed with stage IV PCa present bone metastases when diagnosed [8]. These bone lesions can induce skeletal-related events (SREs) as further complications of the disease, which are experienced by a further 5.9% of the patients [6]. These SREs, like pathological fractures, nerve compression syndromes or hypercalcemia, reduce the quality of life of the patients [9,10,11] and contribute to a major part to the significantly increased mortality and morbidity [12,13,14].

The first publications on the biological effects of diphosphonates, later renamed bisphosphonates, appeared in 1958 [15]. Since then, their importance and use in medicine has increased rapidly, and today, they are applied for treatments of a variety of bone diseases with excessive osteoclast activity (e.g., metastatic and osteolytic bone disease, osteoporosis and Paget’s disease of bone) [16,17].

**Figure 1:**
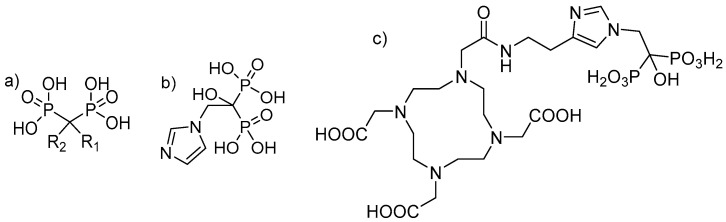
(**a**) Chemical backbone of bisphosphonates. (**b**) Chemical structure of zoledronate with R_1_: OH and R_2_: imidazol-1-ylethyliden. (**c**) Chemical structure of 1,4,7,10-tetraazacyclododecane-1,4,7,10-tetraacetic acid-conjugated zoledronic acid (DOTA-ZOL).

Bisphosphonates are structural analogues of inorganic pyrophosphate (PPi). Although PPi contains two esterified phosphate groups (P-O-P bond) and bisphosphonates link two phosphonate groups covalently to a central carbon (P-C-P bond), they exhibit, to some extent, similar biological properties. In the human metabolism, PPi results from a multitude of reactions as a by-product but also plays a key role in biomineralization pathways [18,19]. Already, in 1962, PPi was found to be responsible for calcification regulation under physiological conditions [20], which formed the basis for further investigations of its role in the calcification of soft tissue, bone mineralisation, metabolism and related clinical disorders [19]. Like PPi, bisphosphonates have a high affinity for hydroxyapatite (HAP) [21,22], one of the main constituents of bone, but being chemically stable and enabling a number of variations in the structure based on the substitutions in the R_1_ and R_2_ positions on the carbon atom (Figure 1) [19].

Since the first medicinal use of a bisphosphonate (etidronate in 1969; first-generation bisphosphonate) as an antiresorptive [17], the pharmacological properties of them have improved significantly. Bisphosphonates of the generation applied today, zoledronate or risedronate, provide a relative antiresorptive potency, which is between 1000- and 10,000-fold higher [23]. Pharmacokinetics are strongly influenced by the two moieties binding to the central carbon. While the two phosphate groups provide the strong affinity for HAP, a hydroxyl group in position R_1_ increases the ability of the bisphosphonate to bind calcium [23,24]. Although this is essential for the affinity to the bone matrix, the antiresorptive potency is dependent on the moiety on R_2_, and a nitrogen or amino group in this position increases the antiresorptive potency relative to first-generation bisphosphonates like etidronate by 10 to 10,000 [23,25]. As shown by some studies, bisphosphonates of the actual generation (nitrogen-containing R_2_) bind to farnesyl pyrophosphate synthase (FPPS) and inhibit its activity [25,26]. The FFPS is a key enzyme in the mevalonic acid pathway (MVAP), and its inhibition correlates with a blockade of the MVAP [27,28]. This intervention leads to a constrained isoprenylation of proteins, which is essential for the regulation of osteoclast activity [29], ultimately inducing osteoclast apoptosis [27,30].

**Figure 2:**
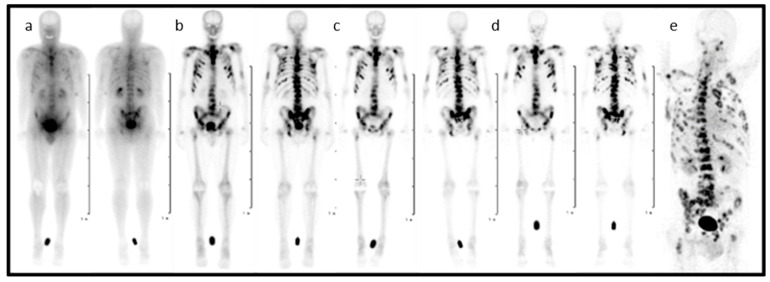
Planar scintigraphy (anterior and posterior) of [^177^Lu]Lu-DOTA-ZOL at different time points (**a**–**d**) and PET/CT of gallium-68 [^68^Ga]Ga-DOTA-ZOL (**e**) in a bronchial carcinoma patient with secondary bone metastases [41].

In the entire process, from diagnosis to the treatment of patients suffering from skeletal metastatic disease, the techniques of nuclear medicine play a key role. ^99m^Tc-labelled bisphosphonates such as methylene diphosphate (MDP) or hydroxy methylene diphosphate (HMDP) have been used in the clinical diagnosis of metastatic bone cancer for many years [31], and therapeutic radionuclides have proven their value in bone pain palliation [32,33,34]. From the current generation of bisphosphonates, zoledronate’s high affinity for hydroxyapatite and its high antiresorptive potency makes it particularly interesting as a targeting vector for theragnostic applications in nuclear medicine. One of the first bisphosphonate derivates suitable for diagnostic imaging as well as therapeutic treatment was (4-{[(bis(phosphonomethyl))carbamoyl]methyl}-7,10-bis(carboxymethyl)-1,4,7,10-tetraazacyclododec-1-yl) acetic acid (BPAMD) [35,36]. BPAMD combines the bisphosphonate with a chelator, namely 1,4,7,10-tetraazacyclododecane-1,4,7,10-tetraacetic acid (DOTA). DOTA forms stable complexes with a variety of medicinal applied radiometals, thus enabling the use of the entire spectrum of nuclear medicine techniques. The first patient studies with gallium-68 ^68^Ga/^177^Lu-labelled BPAMD proved the potential of this theragnostic approach of a DOTA-conjugated bisphosphonate [37,38,39]. This proof-of-principle paved the way for more sophisticated macrocyclic bisphosphonates, employing more potent bisphosphonate basic structures like pamidronate and zoledronate [40], the latter of which already proved its enhanced potential in diagnosis and therapy Figure 2 [41,42].

The presented work arises from the need to implement this novel compound DOTA-conjugated zoledronic acid (ZOL) into radiopharmaceutical routine production prior to patient studies. The transfer of the radiolabelling but particularly the proposed quality control methods exhibited a series of problems and flaws that need to be addressed. On the one hand, the setup of an automated radiolabelling method for gallium-68 using the available module system and its accessories presented some challenges. On the other hand, the quality control was not reliable. This combination of problems led to an extended effort on addressing all these issues. (1) The cassette and consumable kit, as well as the radiolabelling itself, needed to be adjusted accordingly. (2) The need for a purification method using solid-phase extraction (SPE). (3) The bisphosphonate presented issues with the glass reactor vial from the cassette. (4) The known radio thin-layer chromatography (radio TLC) methods could not distinguish between the radiolabelled compound and gallium-68 in its colloidal form. (5) The known radio-TLC method presented a high percentage of false negative results. (6) The known radio-TLC method led to a decomposition of the final compound. (7) The proposed radio high-pressure liquid chromatography (radio HPLC) methods were not reproducible. Despite all efforts, a solution could only be found for a part of the observed challenges, which are described in the following.

## 2. Results and Discussion

The first attempts to implement the novel compound DOTA-ZOL into radiopharmaceutical routine production for intended patient studies exhibited a peck of trouble. An in-depth investigation of the reasons and possible solutions revealed a number of critical points. In the following, the results of the first attempts for the implementation are presented, as well as all findings that are based thereon. For a better explanation and understanding, the radiolabelling/synthesis and purification are divided in two paragraphs and discussed separately.

### 2.1. Radiolabelling I: The Manual Method

**Figure 3:**
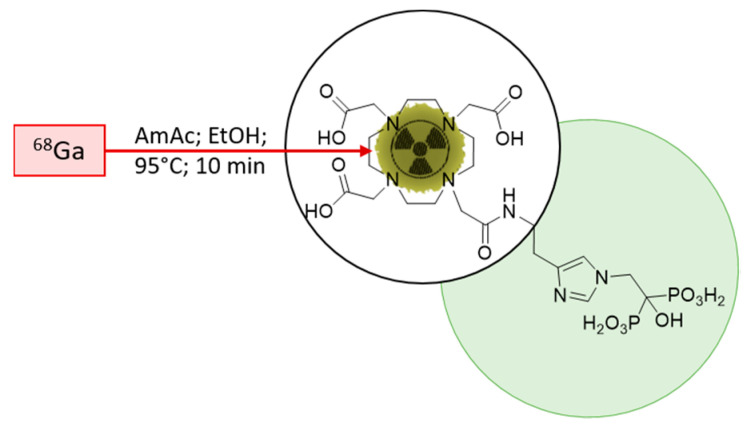
Scheme of the ^68^Ga-radiolabelling of DOTA-ZOL. Highlighted in green, the bisphosphonate moiety and, encircled, the chelating moiety.

The initial starting point for the implementation was the published radiolabelling method by Meckel et al. [40]. To adapt the method for clinical use, the open vial concept was replaced by a closed vial setup, employing a sealed sterile reaction vessel. Consequently, the acetone present in the preparation due to the post-processing of the generator eluate was not evaporated any longer during the reaction. To avoid acetone in general, it was decided to modify the procedure according to Seemann et al. [43]. This radiolabelling method for bisphosphonate takes the advantage of ethanol-based post-processing, which was found to be more suitable in a clinical setting, with the aim to prepare patient doses. A schematic description of the radiolabelling is presented in Figure 3.

Taking this into account resulted in a modified method using 500-µL post-processed [44] gallium-68 with 25 µg (35.6 nmol) of DOTA-ZOL in an ammonium acetate buffer (1 M; pH 4.5) in a sealed, sterile reaction vessel that was agitated at 85 °C for 15 min. The result of the complexation reaction was monitored via radio TLC using the published radio TLC method [40]. For this, an aliquot of the crude product was taken, spotted on the TLC strip and developed in the described solvent system consisting of acetylacetone (acac), acetone (ac) and concentrated hydrochloric acid (HCl) in the ratio 10:10:1.

A series of five consecutive synthesis with starting activities of 441.3 ± 129.4 MBq proved the method to be more unstable than expected. The mean complexation yield (determined in the crude product) was found to be 75.82% ± 25.66%, with an range from 31.9% to 98.9%. Only one of the five synthesis yielded more than 95%. While lacking a suitable purification method, as described later on, the complexation yield of the crude product is equal to the radiochemical purity of the final product. As a result, 80% of the synthesis did not meet the quality control criteria for uncomplexed gallium-68 (<2%) and colloids (<3%) thereof present in the final product. These values were defined based on the pharmacopeial monograph for [^68^Ga]Ga-DOTA-(0)-Phe(1)-Tyr(3))octreotid ([^68^Ga]Ga-DOTA-TOC) [45].

Not only the standard deviation of the complexation yields, especially the wide range from 31.9% to 98.9%, was unforeseen and requested an in-depth investigation. The first revision of the synthesis and its parameters led to an improved method using 50-µg (71.3 nmol) DOTA-ZOL and 90 °C. The modifications resulted in higher complexation yields (mean 85.73% ± 12%), with an range from 68.9% to 96.05% in a series of three consecutive synthesis. Still, the rejection rate (66.6%) was high. Further experiments with elevated temperatures (up to 120 °C) did not improve this result. Surprisingly, the increase of the precursor amount from 25 µg to 50 µg had a lower effect than expected.

With regards to these findings and the unavailability of a purification method, the high rejection rate led to the decision to start all over again, trying to adapt the institutional standard radiolabelling method from a clinical routine for DOTA-ZOL instead of a literature-based method development. Use of this method would have, additionally, the advantage of the availability and grade of the reagents. The second major revision of the synthesis led to the manual method described in the Materials and Methods section, applying 50-µg DOTA-ZOL and sodium chlorine post-processed gallium-68.

In a series of three consecutive synthesis (426.00 ± 87.78 MBq), this method was found to be significantly more reproducible, with a mean complexation yield of 99.56% ± 0.15% determined with the published radio TLC method. The range of the complexation yield was from 99.36% to 99.72%. The radioactivity yield was 73.27% ± 9.87% (n.c.) and the radiochemical yield 87.94% ± 7.99% (d.c.).

Due to this result, it was assumed that the problem was solved and the synthesis under control. Therefore, it was decided to proceed and translate the method on the synthesis module.

### 2.2. Radiolabelling II: First Attempt for Automation

First the available cassette and associated method were modified in terms of purification. As, at this particular time, none of the evaluated SPE methods were found to be suitable and the manual method expected to be reproducible, with complexation yields above 95%, it was decided to eliminate the purification step. The final cassette setup is depicted in Supplemental Figure S1. Using this cassette setup, the manual synthesis was transferred to the module system.

In principle, this was found to be successful. A first series of four consecutive synthesis (427.50 ± 77.87 MBq) revealed a mean complexation yield of 98.27% ± 0.89%, and a range from 97.51% to 99.71% seemed to prove this. Interestingly, with the increasing number of syntheses, it got worse and worse. In a set of 16 syntheses (532 ± 225.50 MBq) under equal reaction conditions, the mean complexation yield was 89.09% ± 11.15%, with a range from 71.07% to 99.71%. From these 16 syntheses, nine did not meet the criteria for quality control (56.3%). Further adjustments in terms of temperature, reaction time, buffer, and precursor amount did not show any effects. A subsequent evaluation with regards to the most common problems with gallium-68 (e.g., metal-contaminated solutions) and the implementation of citric buffer pH 4 as additional radio TLC method, switched the focus of the investigation in the direction of quality control.

### 2.3. Radio TLC Quality Control: Unexpected Outcomes

The radio TLC method applied for determination of the yield of the complexation reaction was taken from literature [40] making use of silica-coated TLC plates as a stationary, and a mixture of acac/ac/conc. HCl (10:10:1) as a mobile, phase. In this solvent system, free uncomplexed gallium-68 is complexed by acetylacetone to [^68^Ga]Ga(acac)_3_ travelling with the solvent front. Gallium-68 in its colloidal form is converted due to the very low pH into ionic [^68^Ga]Ga^3+^, which then also can be complexed and travel with the solvent front. The retention factors (R_f_) for [^68^Ga]Ga-DOTA-ZOL and free, uncomplexed gallium-68, respectively, and ^68^Ga-colloids as an acetylacetone complex, are specified with 0.1 and 0.9, respectively [40].

Retention factors observed during the setup of the automated synthesis for [^68^Ga]Ga(acac)_3_ ranged from 0.5–0.9. Additionally, sometimes, an unusual smear or third peak occurred (Figure S2). To determine the amount of ionic [^68^Ga]Ga^3+^ without ^68^Ga-colloids, as well as to verify the results obtained by the published radio TLC method, the mobile phase was substituted with citric buffer pH 4. The retention factors for [^68^Ga]Ga-DOTA-ZOL, free, uncomplexed gallium-68, respectively, and ^68^Ga-colloids are with 0–0.1, 0.7–1 and 0.1–0.2. Discrimination of the radiopharmaceutical and low amounts of ^68^Ga-colloids is not possible with this method.

In a comparison of the results for both radio TLC methods in terms of free, uncomplexed gallium-68, two observations were made. For the synthesis that would be rejected based on the result of the published method, the particular results always vary widely. Assuming that the amount of activity spotted is similar when applying an aliquot of the same volume from the same preparation, the amount of radiopharmaceuticals detected with the published method is significantly reduced.

Based on these findings, an inspection of the intermediate precision and robustness [46] of the previously published radio TLC method was performed. The survey is described in detail in the Supplementary Materials. The retention factor found for ^68^Ga-colloids was 0.8–1, like published, but for free, uncomplexed gallium-68, an effective value of 0.6–0.9 was found. Additionally, the high concentration of HCl not only converted ^68^Ga-colloids into ionic [^68^Ga]Ga^3+^ travelling with the solvent front. Besides that, it seemed to facilitate the H^+^-assisted dissociation of the DOTA complex [47,48,49].

Assuming that this dissociation in the mobile phase led to the significantly different results of ^68^Ga-contents in both radio TLC methods and, thus, to the incorrect rejection rates, further experiments with [^68^Ga]Ga-DOTA-ZOL were conducted. The incubation of [^68^Ga]Ga-DOTA-ZOL in the mobile phase (acac/ac/conc. HCl (10:10:1)) at room temperature for 5 min resulted in the decomposition of the compound verified by radio TLC with a citric buffer pH 4. Less than 15% of the spotted activity was detected with R_f_ < 0.1, which is assigned to the compound as gallium-68 in its colloidal form, and was not present under these pH conditions. The majority of activity was found at R_f_ 0.5, which was assigned to [^68^Ga]Ga(acac)_3_.

To clarify whether the gallium-68 detected origins from an unintendedly produced outer-sphere complex or from the desired ^68^Ga-DOTA complex, the experiment was repeated with a preparation of [^68^Ga]Ga-DOTA-TOC. To the best of our knowledge, [^68^Ga]Ga-DOTA-TOC did not form stable outer sphere complexes. Therefore, the only source for gallium-68 in a sample of known purity was the ^68^Ga-DOTA complex. Additionally, quality control with radio TLC and radio HPLC of this compound was straightforward and reliable. The experiment confirmed the origin of gallium-68. The majority of activity (>90%) was found at R_f_ 0.5, which was assigned to [^68^Ga]Ga(acac)_3_. The retention factor of [^68^Ga]Ga-DOTA-TOC, initial purity 98%, in citric buffer pH 4 was 0–0.2.

The subsequent prospection for a radio TLC method allowing the determination of free, uncomplexed gallium-68, ^68^Ga-colloids and [^68^Ga]Ga-DOTA-ZOL led to three radio TLC methods found to be suitable to determine the radiochemical purity (Table 1).

A detailed description, as well as images of the corresponding radio TLCs of the here-described experiments, can be found in the Supplementary Materials.

By means of these three radio TLC methods, the high rate of falsely negative results was minimised, and a reliable quality control in terms of the complexation yield was possible. With this amendment, work on the implementation could continue.

### 2.4. Radiolabelling III: Revision of the Methods

Unfortunately, the decomposition of the radiolabelled compound during quality control questioned all previously achieved results for the radiolabelling. Therefore, a revision of the manual and automated method using the augmented radio TLC methods for quality control was executed. The previous results for the mean complexation yield were confirmed in both cases but with significantly reduced deviations. Nevertheless, especially the automated synthesis with a mean complexation yield of ~90% was, without a suitable SPE purification method, still a do-or-die procedure and required a solution.

### 2.5. Solid Phase Extraction: Close By

The simple and fast process of SPE purification is perfectly suitable for radiopharmaceutical needs where time matters. The separation of dissolved or suspended compounds from a mixture results from differences in the physical and chemical properties of the entire species. Based on the properties of the requested compound, it can be retained or pass through the stationary phase of the SPE cartridge during the purification process [50]. Purification of [^68^Ga]Ga-DOTA-ZOL was published using a weak anion exchanger (25 mg, Merck LiChroprep NH_2_), where the product was trapped on and eluted with 2-mL phosphate-buffered saline (PBS) [40].

Based on the published purification method, a SPE cartridge screening was conducted, as the Merck LiChroprep NH_2_ SPE cartridge was not available anymore. In first place, the selection was based on recommendations and suggestions of the technical service of the manufacturers. In second place, after a couple of unexpected and or negative results, tests were extended to every resin type at our disposal. A total of 35 commercially available SPE cartridges were evaluated for their suitability to purify [^68^Ga]Ga-DOTA-ZOL (Table S2). This was achieved using the automated method but with the original cassette setup where the C18 cartridge used for DOTA-peptide radiolabelling was replaced by the entire cartridge. The parameters of interest were trapping, recovery and final purity of the product.

Depending on the trapping behaviour, the cartridges were assigned to four groups: (1) no retention, (2) retention of both species, (3) retention of gallium-68 and (4) retention of the product.

Group 1 included 19 out of these 35 cartridges that did not retain either the product or free, uncomplexed gallium-68. This result was found for the pure reaction mixture containing up to 12 vol% ethanol, as well as for the diluted one containing only 5 vol%. The ethanol content did not affect this result at all. Due to this insufficient trapping performance, these 19 cartridges were dismissed.

Eleven cartridges were assigned to group 2, showing (partial) retention of both species, although not always equally strong. All were dismissed for nonselective trapping and final purity. In the relevant cases, the loss of the product was adjudged too high, with more than 10%, in conjunction with only partial purification.

Group 3 included three cartridges. They retained only gallium-68, while no retention for the product was observable. Two of these three cartridges retained gallium-68 unexpectedly, instead of [^68^Ga]Ga-DOTA-ZOL. Nevertheless, the obtained retention gallium-68 was found to be incomplete in all cases. As a result, in the final product fraction, the amount of free, uncomplexed gallium-68 was reduced compared to the reaction mixture before purification but still present. To overcome this problem, an increased amount of resin should have been the solution. Admittedly, no bigger size was available for two (Chromafix SA and Chromafix HR-XAW) cartridges. For the third cartridge (Chromafix PS-H^+^), the next larger size (L) showed a partial retention of both species and was assigned to group 2. The cartridges of group 3 may be used for purification. Nevertheless, their purifying effect was too low to achieve a product of sufficient quality in terms of free, uncomplexed gallium-68 when the initial amount of it was in a medium or high range. With regards to the mean complexation yield of the automated synthesis, ~90% of the cartridges of group 3 were excluded. The use of these would help in only the minority of syntheses to achieve a releasable product. The majority provided either perfect complexation yields (>95%) or yields with medium or high amounts of uncomplexed gallium-68 left.

The two cartridges in group 4 retained only [^68^Ga]Ga-DOTA-ZOL. While the trapping of the product was nearly quantitative, the purifying effect could not be conclusively evaluated. In both cases, no recovery was achieved. For recovery, in the first place, solvent mixtures predefined as suitable for an injectable radiopharmaceutical were evaluated. Additionally, the supplier-recommended eluents were used. For one cartridge (Strata-X-C polymeric) also, the supplier recommendation did not lead to success; for the other (Chromafix PS-H^+^ (s)), the product was decomposed due to the solvent composition. Both cartridges were found to be unsuitable.

The development of a purification strategy based on only one SPE cartridge was found to be more complicated than expected and hoped. A lack of the presented survey was its incompleteness. The number of commercially available resins or prepacked cartridges is appreciably higher and increases with ongoing research on dedicated materials every year. Besides this, only a single-step purification was evaluated, while a multilevel purification process may be the solution. Despite that, it is a first sight on the issue, indicating the problems due to the structural characteristics of bisphosphonates. It is a balancing act between radiopharmaceutical needs (purity and activity yield) and chemical demands. Nevertheless, if the demand for ^68^Ga-labelled bisphosphonates increases in the future, SPE purifications shall be evaluated in more detail.

### 2.6. Radiolabelling IV: The Journey Goes on

Realising that the SPE purification problem will be a long-term project, the decision was made to figure out which parameters could be optimised to achieve reliable high complexation yields superseding a subsequent purification. From the previous radiolabelling experiments, two findings were scrutinised:

(a) Why is the effect of elevated temperatures low?
(b) Why is the effect of a doubled precursor mass low?

In comparison to other DOTA-conjugated compounds, the effects of these two parameters were too low. Being aware of this moved the chemical properties of zoledronic acid into the focus.

### 2.7. Reactor Screening: Minimal Change, Maximum Impact

Glass vessels are omnipresent in the pharmaceutical industry, as well as radiopharmacy, even though they cannot be considered quite inert; to the contrary, various interactions between glass surfaces and the products could appear [51,52]. One class of pharmaceuticals known to interact with glass containers are bisphosphonates in solutions. These show an interaction with several polyvalent cations present in the glass, leading to an accelerated leaching of the cations and precipitation of the bisphosphonate and, finally, to a shortened drug potency [51]. This interaction is further driven by the pH and/or elevated temperatures. With regards to this, the nanomolar amount of the precursor, as well as a chelator requiring elevated temperatures in combination with a glass reactor, can be assumed to be problematic in a reaction known to be sensitive for metal contaminations and asking for precursor excesses. To avoid those glass-drug interactions in the pharmaceutical industry, special-coated containers are used. The silicon-based coating aims to prevent direct contact of the pharmaceuticals with the glass surfaces and can sustain additional functionalities for further improvements of the surfaces of the container materials.

Based on this, the decision was made to evaluate the reaction vessels in stock for their suitability in radiolabelling DOTA-ZOL. To evaluate the effect of the container on the radiolabelling with gallium-68, two types of special-coated glass vials were compared with the vials in stock under equal radiolabelling conditions using the manual method as described. Reactors from the stock tested were Tc-Elu-15 vials (volume 15 mL; Cis-Bio, Berlin, Germany), as well as the reactors provided with the fluidic kit of the module (volume 10 mL; ABX, Radeberg, Germany). These were compared with SCHOTT Type I plus^®^ with pure SiO_2_ coating and SCHOTT TopLyo^®^ with Si-O-C-H hydrophobic coating (both: volume 10 mL; Schott, Mainz, Germany).

**Figure 4:**
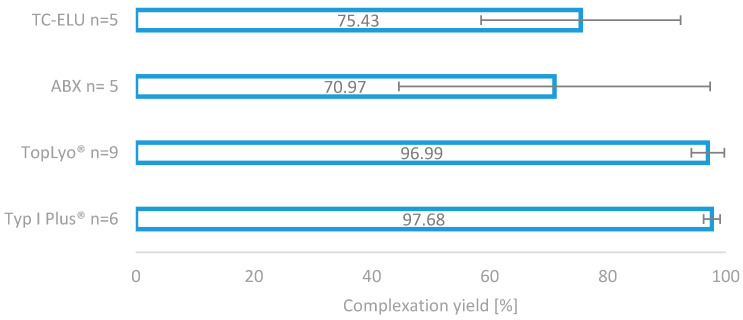
Comparison of the four different reactor vials for radiolabelling. Two normal glass reactors, as well as two silicone-coated glass reactors.

The impressive effect of the silicone coating on the radiolabelling reaction is depicted in Figure 4. Compared to the two glass reactors (TC-ELU-15 75.43% ± 16.92% and ABX 70.9%7 ± 26.38%), the coated vials led to significantly increased and stable complexation yields (TopLyo^®^ 96.99% ± 2.80% and Type I Plus^®^ 97.68% ± 1.39%). While the difference between coated and noncoated vials was evident, no significant effect between the pure silicone coating and hydrophobic Si-O-C-H coating was observable. Both coatings were found to be effective in preventing glass-drug interactions and stabilising the synthesis outcome.

### 2.8. Radiolabelling V: Final Version

With regards to the results of the reactor screening, the manual synthesis was modified in terms of the reaction vial. The high repeatability in combination with complexation yields above 95% facilitated the use of DOTA-ZOL for diagnostic imaging, even without a purification method.

Unfortunately, the coated reactor vials did not fit into the heater of the module system. As the original reactor showed the poorest performance of the four tested reaction vials, the automated method was put on hold until a suitable replacement or a purification method was found. Although the manual method does not meet the demand for clinical routine production, the unfavourable reactor or SPE problem could not be satisfactory addressed; the manual method at least provided [^68^Ga]Ga-DOTA-ZOL in good quality for the first clinical studies.

### 2.9. Radio-HPLC Quality Control

The previously described radio TLC methods provided a fast and reliable way to check the final product for the appearance of gallium-68, either free, uncomplexed or in its colloidal form. Albeit this is very important information in terms of quality control, the technique cannot assess every information needed or wanted, especially for a new investigational compound. This informational gap could be closed by radio HPLC. In a clinical routine, radio HPLC is used to determine not only the content of gallium-68 in ^68^Ga-radiopharmaceuticals but, also, chemical and other radioactive impurities (e.g., radiolysis by-products). A critical requirement, not obtained by radio TLC but by radio HPLC, is the product identification.

The method published for radio HPLC [40] took a detour to take advantage of the standard available C18 columns and water/acetonitrile mixtures as the solvent system frequently used for metal-based radiopharmaceuticals. The aliquot for quality control was incubated with deferoxamine (DFO) to the complex-remaining free gallium-68. So complexed, it was possible to discriminate between [^68^Ga]Ga-DOTA-ZOL and [^68^Ga]Ga-DFO, which was, in contrast to the zoledronate, retained. With this method, the [^68^Ga]Ga-DOTA-ZOL passed the column without retention. Therefore, it was not suitable for an identification of the ^68^Ga-labelled species of interest or the determination of radiolysis products thereof or chemical purity. Additionally, for the theragnostic counterpart [^177^Lu]Lu-DOTA-ZOL, it did not work. Based on this, it was decided to start from scratch again, searching for suitable radio HPLC conditions for [^68^Ga]Ga-DOTA-ZOL.

**Figure 5:**
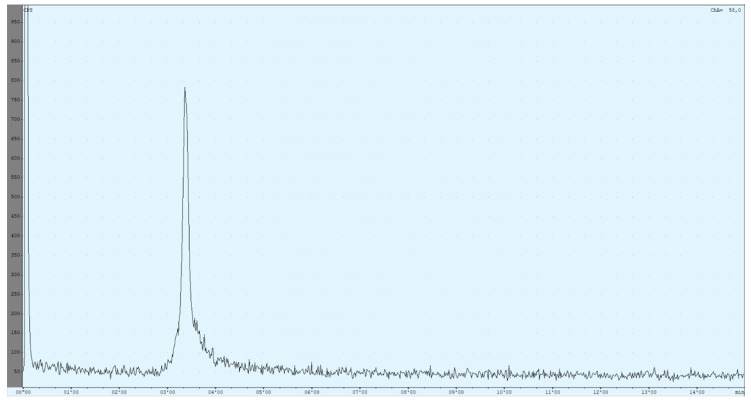
The currently used high-performance liquid chromatography (HPLC) method utilises an isocratic mixture of 90% 59-mM TBAP/10% MeOH (1.2 mL/min). The chromatogram shows the result of a [^68^Ga]Ga-DOTA-ZOL synthesis (complexation yield determined with radio thin-layer chromatography (TLC) was 98%). The first peak refers to all injected activity measured before the column. The ratio between both peaks is nearly 50:50.

A complete list of all evaluated radio HPLC conditions can be found in the Supplemental Materials (Table S3). Finally, a preliminary method was obtained that still has some weaknesses but can be used to identify the product compound. It is based on a method published by Reddy et al. [53] but adapted for [^68^Ga]Ga-DOTA-ZOL (Figure 5).

Due to the setup of the radio HPLC, recovery of the product could be determined immediately. The injected activity passed the radioactivity detector two times, before and after the column. Retention times were determined for free gallium-68 (R_t_ = 2.85 ± 0.03 min), [^68^Ga]Ga-DOTA-ZOL (R_t_ = 3.35 ± 0.01 min), void volume (R_t_ = 2.35 ± 0.04 min) and [^177^Lu]Lu-DOTA-ZOL (R_t_ = 6.30 ± 0.05 min).

The difference in retention times of the different species was very small. Up to now, this problem could not be solved either by changing the flow or the solvent ratio. Additionally, the product peak showed some tailing, which could be improved but not prevented yet. Reduced tailing can be achieved by dilution of the aliquot in an equal amount of the mobile phase before injection. Due to this, only occurring for the product peak, it was assumed that it was induced by the chemical properties of the compound. Further improvements may be achieved by adjusting the pH of the mobile phase.

Nevertheless, the method is not perfect and still requires improvements until a first step is achieved. As the compound shows retention, it is possible to determine it via its retention time directly and not only via the exclusion procedure. This represents an advantage, not only over the previously published method but, also, over the radio TLC. In total, the combination of radio TLC for the determination of the unlabelled ^68^Ga-species and radio HPLC for product identification and chemical purity empowers a more reliable quality control.

## 3. Materials and Methods

### 3.1. Radiolabelling

Gallium-68 was obtained from a ^68^Ge/^68^Ga-generator (iThemba Labs, Cape town, South Africa). Synthesis was performed utilising an automated cassette module (GAIA; Elysia-Raytest, Straubenhardt, Germany). Standard fluidic and reagent kit for ^68^Ga-radiolabelling of peptides (ABX advanced biochemical compounds; GmbH, Radeberg, Germany) were used. The standard strong cation exchanger (SCX) provided by the reagent kit was replaced by 200-mg STRATA SCX (Phenomenex, Torrance, CA, USA). All reagents used were from the reagent kit unless otherwise stated. TraceSelect water, as well as ethanol Ph. Eur., were purchased from Merck (Darmstadt, Germany). DOTA-ZOL, synthesised according to the literature [40], was obtained from CheMatech (CheMatech, Dijon, France) and diluted with TraceSelect water to a final concentration of 1 mg/mL.

Manual synthesis was performed by adding gallium-68 in 500-µL eluent to 60-µL DOTA-ZOL in 4.8-mL 0.08-M ammonium acetate buffer (pH 4.5) and 500-µL ethanol in a sealed reactor. After radiolabelling in a thermo-shaker (Hettich-Benelux, Geldermalsen, Netherlands) at 95 ± 4.5 °C for 600 s, an aliquot of the reaction mixture was analysed to investigate the chelate formation.

Reactors tested for radiolabelling were TYP I plus and TopLyo obtained from Schott (Mainz, Germany), the reactor provided with the fluidic kit of the module (ABX; Radeberg, Germany), as well as standard Tc-Elu-15 vials from Cis-Bio (Berlin, Germany). All reactors were tested under equal radiolabelling conditions using the manual method.

Automated synthesis was performed using the standard fluidic kit without C18 purification (Figure S1). Gallium-68 in 450-µL eluent were added to 3.28-mL 0.08-M ammonium acetate buffer (pH 4.5) and 500-µL ethanol. After radiolabelling at 95 ± 4.5 °C for 648 ± 112 s, the reaction mixture was diluted with isotonic saline solution and sterile-filtered into the final product vial. Analysis of the complexation was performed on an aliquot of the final product solution.

SPE cartridges tested for purification of [^68^Ga]Ga-DOTA-ZOL are listed in detail in the Supplementary Materials (Table S2). Preconditioning of the particular column was performed according to the manufacturer’s instructions. To evaluate the different SPE columns for the automated method but with the original cassette setup trap, either [^68^Ga]Ga-DOTA-ZOL or free, uncomplexed gallium-68 was used on the respective column. For all experiments, either the pure or diluted reaction mixture was passed over the entire cartridge. Recovery was evaluated using the data from the module providing 4 radioactivity detectors. Additional manual synthesis was performed in cases were only one species was retained. To obtain a product suitable for medical use, only ethanol, water, saline or mixtures thereof were considered as solvents for elution. Complexation yield and purity were quantified via radio TLC using the methods described in Table 1 utilising aliquots of each the pure reaction mixture, waste and eluate from the cartridge. Rejection criteria were: no selective trapping of one species, loss of product during purification steps >10% and purification effect not sufficient.

### 3.2. Quality Control

Solvents and chemicals for quality control are listed below. Optigrade acetonitrile (ACN) was obtained from PromoChem (LGC Standards; GmbH, Wesel, Germany); acetylacetone (acac) from Sigma-Aldrich (St. Louis, MO, USA); water for chromatography (LC-MS Grade) LiChrosolv, Certipur citric buffer pH 4, Certipur citric buffer pH 5, acetone (ac), methanol (MeOH), hydrochloric acid (HCl) suprapure 30% and trifluoroacetic acid (TFA) from Merck (KGaA, Darmstadt, Germany); ethylacetate (EtOAc) from ACROS Organics (Geel, Belgium) and saline solution from Fresenius Kabi (Bad Homburg, Germany). Triethylammonium phosphate (TEAP) solution for HPLC, buffer solution 1 M, pH 3.0 (Merck KGaA, Darmstadt, Germany). Tri-sodium citrate dihydrate for analysis EMSURE^®^ 29.4 g (100 mMolar) (Merck KGaA, Darmstadt, Germany). Sodium phosphate 96% 16.3 g (100 mMolar) (Merck KGaA, Darmstadt, Germany). The 60-mM tetrabutylammonium phosphate buffer (TBAP) was prepared by dissolution of 40.0 g (0.294 mol) of dipotassium dihydrogen phosphate (Merck KGaA, Darmstadt, Germany) and 20.0 g (0.059 mol) of tetrabutylammonium hydrogensulfate (ACROS Organics, Geel, Belgium) in 1000 mL of water. Unless otherwise indicated, this solution was used for analyses.

For quality control, an aliquot of about 150 µL was taken from the reaction mixture (manual method) or from the final formulation (automated method). Radioactivity was measured with a dose calibrator (ISOMED 2010; MED Nuklear-Medizintechnik Dresden GmbH, Dresden, Germany).

Radio thin-layer chromatography (radio TLC) was analysed with a single trace radio TLC scanner (PET miniGITA; Elysia-Raytest, Straubenhardt, Germany) and an evaluation software (GinaStar TLC; Elysia-Raytest, Straubenhardt, Germany). A series of stationary phases were evaluated with a variety of different mobile phases, which are listed in detail in the Supplemental Materials (Table S1).

Radio-HPLC was performed using Agilent 1260 Infinity II reverse-phase HPLC system (Agilent Technologies, Santa Clara, CA, USA) equipped with GABI γ-HPLC flow detector (Elysia-raytest, Straubenhardt, Germany) and a PC interface running Gina Star software (Elysia-raytest, Straubenhardt, Germany). All columns evaluated, as well as the mobile phases and gradient systems, are listed in detail in the Supplemental Materials (Table S3).

## 4. Conclusions

The implementation of the novel radiopharmaceutical [^68^Ga]Ga-DOTA-ZOL into clinical routine production exhibited more challenges than expected for a previously published compound. For all of these revealed issues, an intensive analysis for possible solutions was performed. Not every issue could be addressed to our entire satisfaction (e.g., still, the automated method is not suitable for routine production due to the lack of proper coated reactors) or even solved at all (e.g., SPE purification still not possible without a high loss of activity). Nevertheless, the previously published radio TLC method could be improved and provides reliable information about the complexation yield in combination with two other (one newly developed) methods. Additionally, as a first step forward, a radio HPLC identification was successfully made, even though there is still room and need for improvement. Further enhancements may be achieved by optimising the pH, as well as further adjustments in the solvent composition and/or flow rate, and are the focus of ongoing research.

In conclusion, the example of the DOTA-conjugated zoledronate showed that, sometimes, very little changes lead to a high impact on radiolabelling outcomes (e.g., normal glass reaction vessels vs. coated reaction vessels).

## Figures and Tables

**Table 1:** List of the published (first row) and the finally used radio thin-layer chromatography (TLC) methods.

Mobile Phase	R_f_ Compound	R_f_ [^68^Ga]Ga^3+^	R_f_^68^Ga-Colloids
acac/ac/HCl (1:1:0.1) [40]	0–0.2	0.6–0.9	0.8–1
acac/ac (1:1)	0–0.1	0.7–0.8	0–0.1/0.5–0.9
Citric buffer pH 4	0–0.1	0.7–1	0.1–0.2
TBAP/MeOH (9:1)	0.7–0.8	0.1–0.3	0.1–0.2

## Supplementary Materials

The following are available online: Figure S1: Schematic illustration of the fluidic kit setup of the automated synthesis of [^68^Ga]Ga-DOTA-ZOL. Figure S2: Radio TLC chromatograms of the different species potentially present in a [^68^Ga]Ga-DOTA-ZOL synthesis developed in acac/ac/conc. HCl (10:10:1) with silica 60 F254 TLC plates as the stationary phase. Appearance in the following order: final product of a [^68^Ga]Ga-DOTA-ZOL synthesis, ^68^Ga-colloids and [^68^Ga]Ga^3+^. Figure S3: Radio TLC chromatograms of the different species potentially present in a [^68^Ga]Ga-DOTA-ZOL synthesis developed in acac/ac with silica 60 F254 TLC plates as the stationary phase. Appearance in the following order: final product of a [^68^Ga]Ga-DOTA-ZOL synthesis, ^68^Ga-colloids and [^68^Ga]Ga^3+^. Figure S4: Radio TLC chromatograms of the different species potentially present in a [^68^Ga]Ga-DOTA-ZOL synthesis developed in citric buffer pH 4 with silica 60 F254 TLC plates as the stationary phase. Appearance in the following order: final product of a [^68^Ga]Ga-DOTA-ZOL synthesis, ^68^Ga-colloids and [^68^Ga]Ga3+ present as the citrate complex. Figure S5: Radio TLC chromatograms of the different species potentially present in a [^68^Ga]Ga-DOTA-ZOL synthesis developed in TBAP/MeOH (9:1) with silica 60 F254 TLC plates as the stationary phase. Appearance in the following order: final product of a [^68^Ga]Ga-DOTA-ZOL synthesis, ^68^Ga-colloids and [^68^Ga]Ga^3+^. Figure S6: Change of composition during incubation in the solvent system of the published radio TLC method developed in citric buffer pH 4 with silica 60 F254 TLC plates as the stationary phase. First row: radio TLC chromatogram of [^68^Ga]Ga-DOTA-ZOL after synthesis. Second row: [^68^Ga]Ga-DOTA-ZOL incubated 5 min in a preparation of acac/ac/conc. HCl (10:10:1). Third row: ^68^Ga eluate incubated 5 min in a preparation of acac/ac/conc. HCl (10:10:1). Fourth row: [^68^Ga]Ga-DOTA-TOC (initial complexation rate 98%) after incubation in a preparation of acac/ac/conc. HCl (10:10:1). Table S1: List of investigated radio TLC conditions. Greyed cells represent no single peak or smear of the species. Table S2: List of all evaluated SPE cartridges. Table S3: List of investigated radio HPLC conditions. Greyed cells represent no successful discrimination.
