# Supplementary material for: DOTA-ZOL: A Promising Tool in Diagnosis and Palliative Therapy of Bone Metastasis—Challenges and Critical Points in Implementation into Clinical Routine
Source: Molecules. 2020 Jun 30;25(13):2988. doi: 10.3390/molecules25132988 (PMC7412164; doi:10.3390/molecules25132988)
Supplement: Supplementary file 1 [file molecules-25-02988-s001.pdf]

Supplementary Materials

Dota-Zol: A Promising Tool in Diagnosis and Palliative Therapy of Bone Metastasis—Challenges and Critical Points in Implementation into Clinical Routine

Michael Meisenheimer [1], Stefan Kürpig [1], Markus Essler [1] and Elisabeth Eppard [2,*]

[1] Department of Nuclear Medicine, University Hospital Bonn, D-53127 Bonn, Germany; michael.meisenheimer@ukbonn.de (M.M.); stefan.kuerpig@ukbonn.de (S.K.); markus.essler@ukbonn.de (M.E.)

[2] Positronpharma SA, Rancagua 878, Providencia 7500921, Chile

* Correspondence: eeppard@positronpharma.cl

Academic Editor: Diego Muñoz-Torrero

Received: 11 June 2020; Accepted: 19 June 2020; Published: 30 June 2020

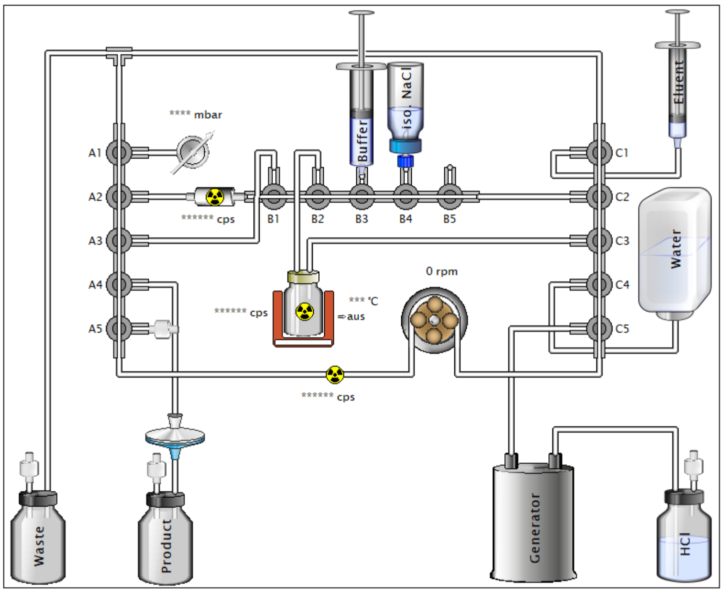

Figure S1. Schematic illustration of the fluidic kit setup of the automated synthesis of [^{68}Ga]Ga-DOTA-ZOL.

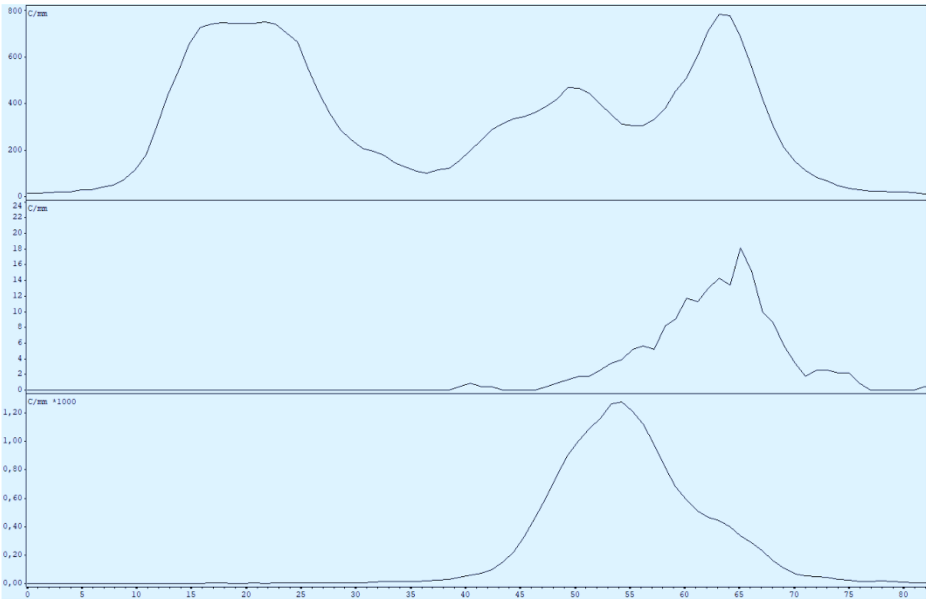

Figure S2. Radio-TLC chromatograms of the different species potentially present in a [68Ga]Ga-DOTA-ZOL synthesis developed in acac/ac/conc. HCl (10:10:1) with silica 60 F254 TLC plates as stationary phase. Appearance in the following order: final product of a [68Ga]Ga-DOTA-ZOL synthesis, 68Ga-colloids, [68Ga]Ga^{3+}.

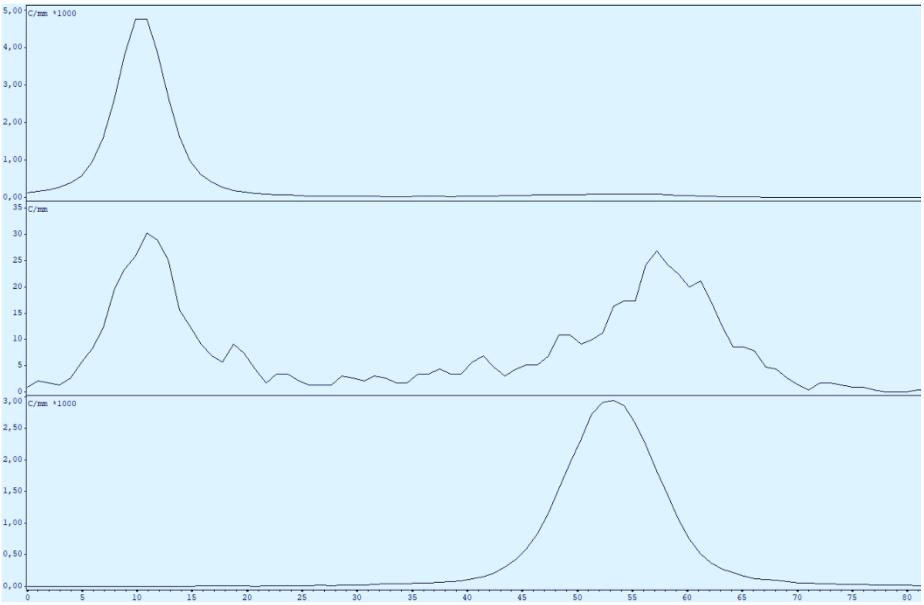

Figure S3. Radio-TLC chromatograms of the different species potentially present in a [68Ga]Ga-DOTA-ZOL synthesis developed in acac/ac with silica 60 F254 TLC plates as stationary phase. Appearance in the following order: final product of a [68Ga]Ga-DOTA-ZOL synthesis, 68Ga-colloids, [68Ga]Ga^{3+}.

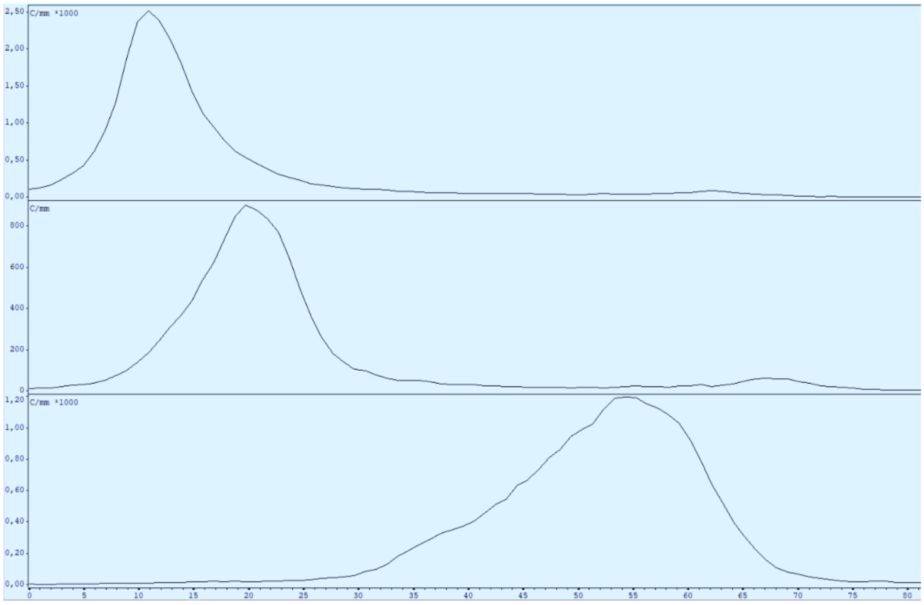

Figure S4. Radio-TLC chromatograms of the different species potentially present in a [^{68}Ga]Ga-DOTA-ZOL synthesis developed in citric buffer pH 4 with silica 60 F254 TLC plates as stationary phase. Appearance in the following order final product of a [^{68}Ga]Ga-DOTA-ZOL synthesis, ^{68}Ga-colloids, [^{68}Ga]Ga3+ present as citrate complex.

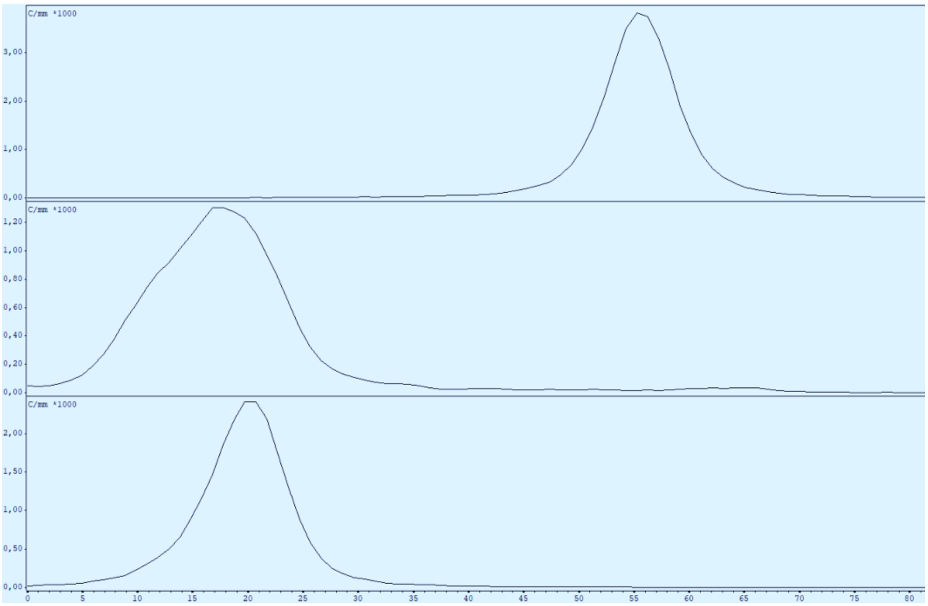

Figure S5. Radio-TLC chromatograms of the different species potentially present in a [^{68}Ga]Ga-DOTA-ZOL synthesis developed in TBAP/MeOH (9:1) with silica 60 F254 TLC plates as stationary phase. Appearance in the following order final product of a [^{68}Ga]Ga-DOTA-ZOL synthesis, ^{68}Ga-colloids, [^{68}Ga]Ga^{3+}.

Table S1. List of investigated radio-TLC conditions. Greyed cells represent no single peak or smear of the species.

Mobile phase	Stationary phase					
	TLC silica 60 F254			iTLC SG		
	R_f A	R_f B	R_f C	R_f A	R_f B	R_f C
0.9% saline						
ac						
acac	0-0.2	0.8-1				

acac/ac (1:1)	0-0.1	0.7-0.8			
acac/ac/HCl (1:1:0.1) [1]		0.6-0.9	0.8-1		
ACN				0-0.2	0.8-1
Citric buffer pH 4	0-0.1	0.7-1	0.1-0.2		
Citric buffer pH 5	0-0.1	0.7-1	0.1-0.2		
EtOAc					
H_2O					
MEK					
MeOH					
PrOH					
TBAP	0.7-1				
TBAP/ac (1:1)					
TBAP/acac (1:1)					
TBAP/acac (3:1)					
TBAP/MeOH (9:1)	0.7-0.8	0.1-0.3	0.1-0.2		

A: [^{68}Ga]Ga-DOTA-ZOL; B: [^{68}Ga]Ga^{3+}; C: ^{68}Ga-colloids.

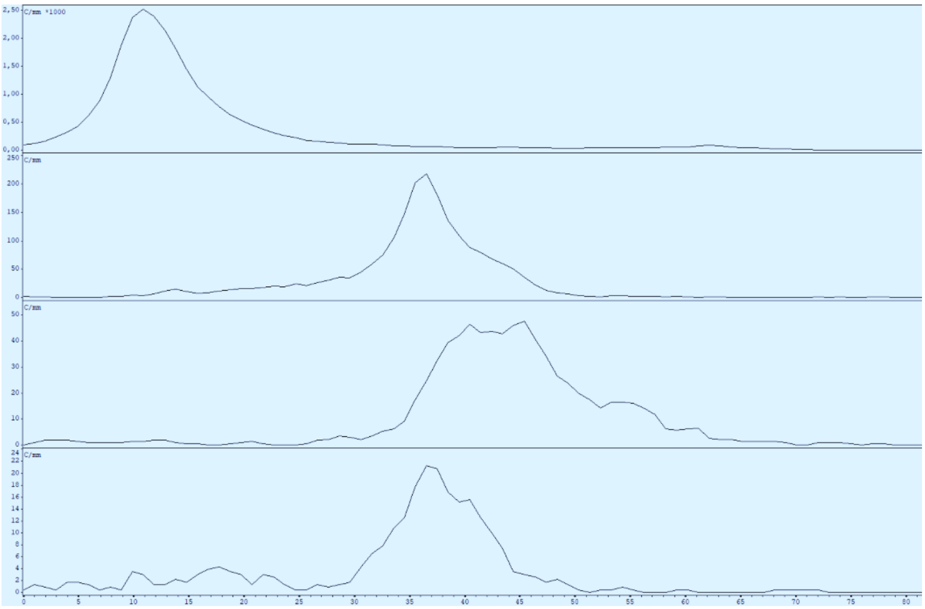

Figure S6. Change of composition during incubation in the solvent system of the published radio-TLC method developed in citric buffer pH 4 with silica 60 F254 TLC plates as stationary phase. First row: radioTLC chromatogram of [^{68}Ga]Ga-DOTA-ZOL after synthesis. Second row: [^{68}Ga]Ga-DOTA-ZOL incubated 5 min in a preparation of acac/ac/conc. HCl (10:10:1). Third row: ^{68}Ga eluate incubated 5 min in a preparation of acac/ac/conc. HCl (10:10:1). Fourth row: [^{68}Ga]Ga-DOTA-TOC (initial complexation rate 98 %) after 5 min incubation in a preparation of acac/ac/conc. HCl (10:10:1).

Table S2. List of all evaluated SPE cartridges.

	SPE Cartridge	Size	Should Retain	Retain	Comments
	**Agilent**				
1	Bond Elut SCX	500 mg	Ga	Ga; P	Dismissed
2	Bond Elut Certify	200 mg	Ga	-	Dismissed
	**Grace**				
3	Alltech Extract-Clean IC-Ag	1.5 ml	P	Ga; P	Dismissed
4	Alltech Extract-Clean IC-H	0.5 ml	P	-	Dismissed
5	Alltech Extract-Clean IC-OH	1.5 ml	P	Ga; P	Dismissed

	**Macherey-Nagel**				
6	Chromabond HILIC	500 mg	P	-	Dismissed
7	Chromabond HR-XC	60 mg	P	-	Dismissed
8	Chromafix HR-XC	S	P	Ga; P	Dismissed
9	Chromafix C18 ec	S	P	-	Dismissed
10	Chromafix C4	M	P	-	Dismissed
11	Chromafix HR-P	M	P	-	Dismissed
12	Chromafix HR-P	L	P	-	Dismissed
13	Chromafix HR-XA	S	P	Ga; P	Dismissed
14	Chromafix HR-XAW	S	P	-	Dismissed
15	Chromafix HR-XAW	M	P	Ga	Partial purification
16	Chromafix PS-Ag^+	L	P	Ga; P	Dismissed
17	Chromafix PS-BA	M	P	-	Dismissed
18	Chromafix PS-H^+	S	Ga	P	No recovery
19	Chromafix PS-H^+	M	Ga	Ga	Partial purification
20	Chromafix PS-H^+	L	Ga	Ga; P	Dismissed
21	Chromafix PS-OH-	M	P	Ga; P	Dismissed
22	Chromafix SA	M	P	Ga	Partial purification
23	Chromafix SB	M	P	Ga; P	Dismissed
	**Waters**				
24	Sep-Pak Alumina N Plus	280 mg	P	-	Dismissed
25	Sep-Pak C18 Plus	360 mg	P	Ga; P	Dismissed
26	Sep-Pak C18 Plus Light	130 mg	P	-	Dismissed
27	Sep-Pak Accell Plus QMA Plus	360 mg	P	-	Dismissed
28	Sep-Pak Accell Plus QMA Plus Light	130 mg	P	-	Dismissed
29	Sep-Pak Silica Plus	690 mg	P	-	Dismissed
30	Sep-Pak Silica Plus Light	120 mg	P	-	Dismissed
31	OASIS MCX 3 cc Vac	60 mg	P	-	Dismissed
32	OASIS MCX 6 cc Vac	150 mg	P	-	Dismissed
	**Phenomenex**				
33	Strata SCX	200 mg	Ga	Ga; P	Dismissed
34	Strata-X-C	200 mg	P	-	Dismissed
35	Strata-X-C polymeric	200 mg	P	P	No recovery

P: $[^{68}Ga]$Ga-DOTA-ZOL.

Table 3. List of investigated radio-HPLC conditions. Greyed cells represent no successful discrimination.

**Mobile Phase**	**Column**										
	A	B	C	D	E	F	G	H	I	J	K
TBAP[a]											
TBAP/MeOH 95:5 [a*]											
TBAP/MeOH 90:10[a*]							✓				
TBAP/MeOH 85:15[a*]											
TBAP/MeOH 75:25[b*]											
100 mM Na_3PO_4/100 mM $Na_3C_6H_5O_7$ pH 4.5[c]											
10 mM TBA-citrate pH 4.5/ACN[d]											
100 mM TEAP pH 2.24[a]											
0.1 % TFA in H_2O/0.1 % TFA in ACN[e]											
0.1 % TFA in H_2O/0.1 % TFA in ACN[f]											

[a]isocratic, flow 0.5; 0.7; 1; 1.2 (in 15 min) and 1.5 ml/min; in 25 min. [b]isocratic, flow 0.7; 1 and 1.2 ml/min; in 25 min. [c]gradient A:B (0:100) → A:B (0:100) in 30 min, flow 0.7; 1.2 ml/min, (provided by ITG, Germany). [d]gradient A:B (70:30) → A:B (20:80) in 30 min, flow 0.7; 1.2 ml/min, (provided by ITG,

Germany). [e]gradient A:B (95:5) → A:B (20:80) in 25min, flow 0.7; 1.2 ml/min [1] [f]gradient A:B (100:0) → A:B (0:100) in 20min → A:B (50:50) from 20 to 25min, flow 0.7 ml/m.in
